# Comparison of tick-borne pathogen prevalence in *Ixodes ricinus* ticks collected in urban areas of Europe

**DOI:** 10.1038/s41598-020-63883-y

**Published:** 2020-04-24

**Authors:** Anna Grochowska, Robert Milewski, Sławomir Pancewicz, Justyna Dunaj, Piotr Czupryna, Anna Justyna Milewska, Magdalena Róg-Makal, Sambor Grygorczuk, Anna Moniuszko-Malinowska

**Affiliations:** 1 Department of Infectious Diseases and Neuroinfections, Medical University of Białystok, Żurawia 14, 15-540 Białystok, Poland; 2 Department of Statistics and Medical Informatics, Medical University of Białystok, Szpitalna 37, 15-295 Białystok, Poland; 3 Department of Invasive Cardiology, Medical University of Białystok, M. Skłodowskiej-Curie 24 A, 15-276 Białystok, Poland

**Keywords:** Parasitic infection, Epidemiology

## Abstract

Tick-borne diseases are a major threat to human and animal health. An increasing number of natural habitats have been transformed into urban areas by human activity; hence, the number of reported tick bites in urban and suburban areas has risen. This retrospective analysis evaluated 53 scientific reports concerning infections of *Ixodes ricinus* ticks collected from urban and suburban areas of Europe between 1991 and 2017. The results indicate significant differences in many variables, including a higher number of *Anaplasma phagocytophilum* infections in Eastern Europe than in Western Europe. The opposite result was observed for *Candidatus* Neoehrlichia mikurensis infections. A comparison of climate zones revealed that *Borrelia burgdorferi* s.l. infections have the greatest median incidence rate in subtropical climate zones. No statistical significance was found when comparing other tick-borne pathogens (TBPs), such as *Borrelia miyamotoi, Rickettsia* spp., *Babesia* spp., *Bartonella* spp., *Ehrlichia* spp., *Coxiella burnetii* and *Francisella tularensis*. The analysis also showed significant differences in the overall prevalence of TBPs according to average temperatures and rainfall across Europe. This retrospective study contributes to the knowledge on the occurrence and prevalence of TBPs in urbanized areas of Europe and their dependence on the habitats and geographical distributions of ticks. Due to the increased risk of tick bites, it is of great importance to investigate infections in ticks from urban and suburban areas.

## Introduction

Tick-borne diseases (TBDs) are an emerging health problem for humans and domestic and farm animals^1^. The most common and well-known disease in the USA and Europe is Lyme borreliosis, a systemic infectious disease caused by *Borrelia burgdorferi* sensu lato (s.l.) spirochetes transmitted by ticks mainly from the genus *Ixodes*^1–4^. The incidence rate of Lyme borreliosis has increased significantly over the last few decades, both in the USA and Europe^2^. Moreover, with the additional detection of new pathogens that cause less well known infections, such as anaplasmosis, babesiosis or rickettsiosis, TBDs are attracting ever-growing interest^3^.

In the USA, approximately 30 000 cases of Lyme borreliosis are reported annually, although recent reports indicate that the actual number of infections amounts to 300 000 cases^5,6^.

The disease is also widespread in Europe. Nevertheless, no accurate data on the incidence rate are available due to the lack of a homogenous reporting system^5^. It is estimated that there are approximately 65 400 cases per year^2^. The highest incidence rates occur in Germany, Austria, Slovenia and Sweden^5^.

There are many factors contributing to the increase in TBD incidence. The most common is global warming. Another major risk factor for humans is the transformation of natural ecosystems into cities or recreational areas due to urbanization, forcing plants and animals to leave their habitats or adapt to the new environment^7^. Ticks are an example of species that adapt to new conditions; hence, an increasing number of tick bites in urban and suburban areas, such as city parks or suburban forests, are reported. Current statistics show that more than half of the world’s population lives in urban areas^4^. Over the last few decades, the subject of infections in ticks collected from urban and suburban areas has been gathering increasing interest among researchers. Akimov and Nebogatkin showed that between 1907 and 2014, the number of publications on ticks in urban landscapes increased almost ten-fold. They also point out that the growing interest is related to the appearance of new diagnostic methods and less well known tick-borne infections, such as babesiosis or rickettsiosis^7^.

The literature review presented in the current study showed that the number of scientific reports summarizing data regarding infections in ticks from urban surroundings is scarce. For this reason, we decided to attempt to compile the available data and support the analysis with statistical methods.

The aim of our research was to compare the prevalence rates of various pathogens in *Ixodes ricinus* ticks collected from urban and suburban areas of Europe according to climate zone, average temperature and rainfall in January and July. All the pathogens detected in *I. ricinus* were analysed in two ways: individually and for overall prevalence (i.e., all the pathogens detected in *I. ricinus*).

## Materials and methods

A comprehensive PubMed literature search was conducted. Reports were included in the assessment if they provided information regarding infections in *I. ricinus* ticks collected from urban and suburban areas in Europe. All the ticks were collected using the dragging method and analysed with PCR. Publications in which results were given in pools were excluded because it was impossible to obtain a specific number of infected ticks. Fifty-three scientific reports from 1991 to 2017 met the inclusion criteria^1,8–59^ (see Supplementary Table S1). To establish whether pathogen prevalence depends on climate conditions or geographical location, the data were divided into the following categories for statistical analysis:

- Europe – Eastern and Western
- Europe – Northern and Southern
- Climate zone – temperate warm climate, temperate cold climate, and subtropical climate
- Average January temperatures – above and below 0 °C
- Average July temperatures – above and below 20 °C
- Average January rainfall – above and below 50 mm
- Average July rainfall – above and below 50 mm.

The climate condition categories were chosen because of tick sensitivity to temperature and humidity^60–62^, which may affect the questing activity of ticks and transmission rates of pathogens to hosts, including humans. The geographical division of Europe was established according to the United Nations Statistic Division^63^. Statistical analysis was performed using the Statistica 12.0 program (StatSoft, USA). The prevalence of pathogens in the European regions (northern, southern, eastern, western) as well as average temperatures and rainfall were calculated using a Mann-Whitney test. A Kruskal-Wallis test was used for the assessment of pathogen prevalence in relation to the climate zones. All the tests were carried out individually for every pathogen detected in *I. ricinus* ticks, as well as for overall tick-borne pathogen (TBP) prevalence. For all the tests, the threshold for statistical significance was p < 0.05.

## Results

The data obtained from the literature were compiled and are presented in Supplementary Table S1, divided into countries, cities, number of examined ticks and percentage of detected pathogens (individual and overall) (see Supplementary Table S1). The comparative analysis showed statistically significant differences in the following categories. Out of all the tested pathogens, *B. burgdorferi* s.l., *Borrelia afzelii*, *Anaplasma phagocytophilum* and *Candidatus* Neoehrlichia mikurensis significantly differed in at least one category. Other TBPs, such as *Borrelia miyamotoi, Rickettsia* spp., *Babesia* spp., *Bartonella* spp., *Ehrlichia* spp., *Coxiella burnetii* and *Francisella tularensis*, did not show statistical significance (see Supplementary Table S1). However, all the detected pathogens were included in the analysis of overall TBP prevalence.

### Tick-borne pathogen prevalence related to European region, average temperatures and rainfall

#### Southern and Northern Europe

The comparative analysis showed no statistically significant differences between Northern and Southern Europe.

#### Western and Eastern Europe

**Figure 1:**
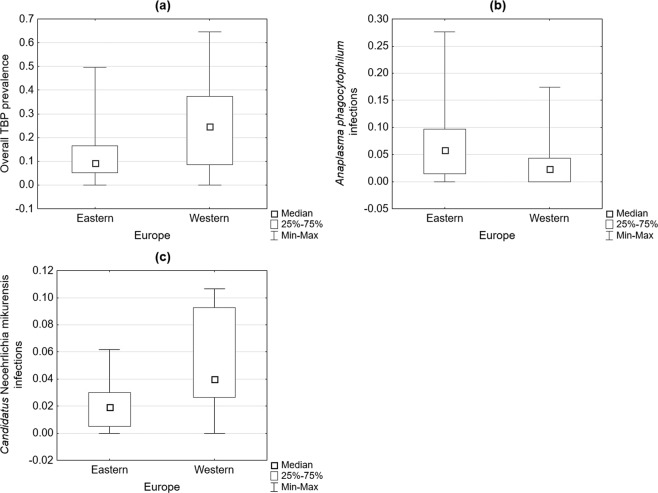
Differences in the overall tick-borne pathogen (TBP) prevalence (**a**), *Anaplasma phagocytophilum* infection rates (**b**) and *Candidatus* Neoehrlichia mikurensis infection rates (**c**) depending on geographical region (Western and Eastern Europe).

The comparative analysis of different variables in Western and Eastern Europe revealed several statistically significant differences (Table 1). The overall TBP prevalence and *Ca*. Neoehrlichia mikurensis infection rate were greater in Western Europe, but in the case of *A. phagocytophilum*, the median incidence rate was higher in Eastern Europe (Fig. 1).

**Table 1:** Comparison of the overall tick-borne pathogen (TBP) prevalence and *Anaplasma phagocytophilum*, *Candidatus* Neoehrlichia mikurensis and *Borrelia burgdorferi* s.l. infections in *Ixodes ricinus* ticks according to European region, average temperatures and rainfall.

Category	Variable	p value
Europe: Western and Eastern	Overall TBP prevalence	0.001
	*A. phagocytophilum* infections	0.011
	*Ca*. Neoehrlichia mikurensis infections	0.019
Average January temperatures: above and below 0 °C	Overall TBP prevalence	0.026
Average July temperatures: above and below 20 °C	Overall TBP prevalence	0.001
	*B. burgdorferi* s.l. infections	0.001
Average January rainfall: above and below 50 mm	Overall TBP prevalence	0.003
Average July rainfall: above and below 50 mm	Overall TBP prevalence	0.001

#### Average January temperatures

**Figure 2:**
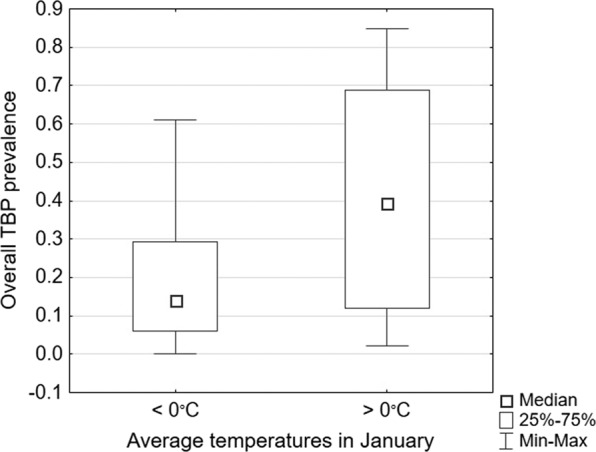
Differences in the overall tick-borne pathogen (TBP) prevalence depending on average January temperatures.

The comparative analysis of variables depending on the average temperature in January (above and below 0 °C) showed statistically significant differences in the overall TBP prevalence, with a higher median incidence rate in regions with average temperatures above 0 °C (Table 1, Fig. 2).

#### Average July temperatures

**Figure 3:**
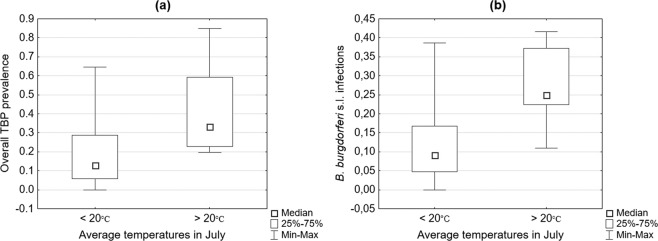
Differences in overall tick-borne pathogen (TBP) (**a**) and *Borrelia burgdorferi* s.l. infection rates (**b**) depending on average July temperatures.

In this category, the comparative analysis revealed statistically significant differences in the overall TBP prevalence and *B. burgdorferi* s.l. infections, with greater median incidence rates in the areas with average temperatures above 20 °C (Table 1, Fig. 3).

#### Average January rainfall

**Figure 4:**
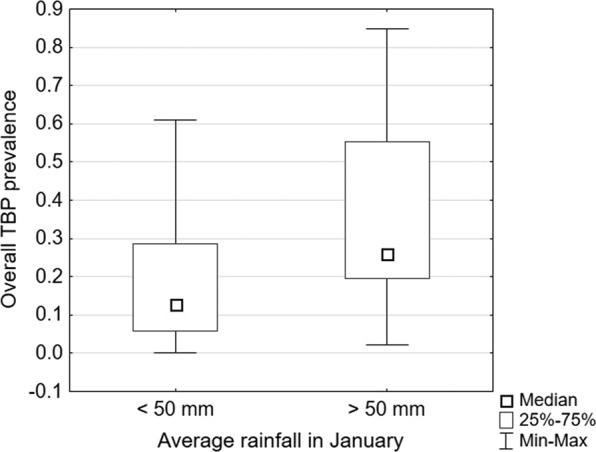
Difference in the overall tick-borne pathogen (TBP) prevalence depending on average January rainfall.

The analyses according to average January rainfall revealed regional differences, with statistically significant differences in the overall TBP prevalence and greater median incidence rates in the areas with rainfall above 50 mm (Table 1, Fig. 4).

#### Average July rainfall

**Figure 5:**
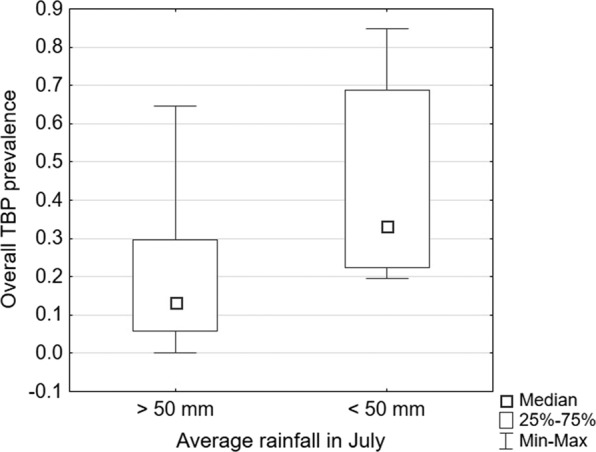
Differences in the overall tick-borne pathogen (TBP) prevalence depending on average July rainfall.

The comparative analysis showed statistically significant differences in the overall TBP prevalence, with greater median incidence rates in regions with lower rainfall levels (below 50 mm) (Table 1, Fig. 5).

### Tick-borne pathogen prevalence depending on climatic zone

**Figure 6:**
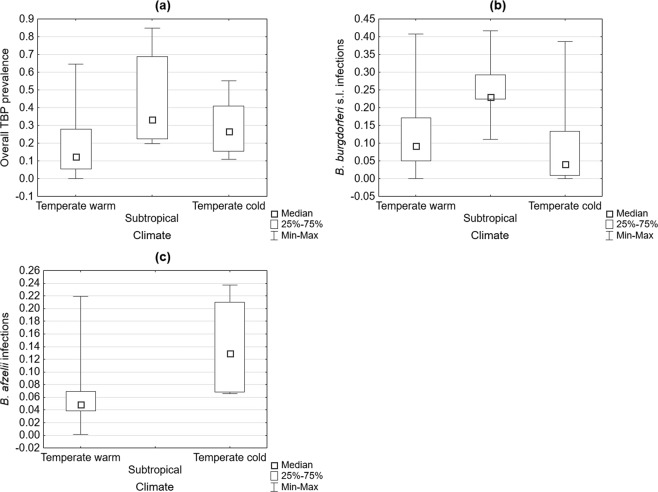
Differences in the overall tick-borne pathogen (TBP) prevalence (**a**), *Borrelia burgdorferi* s.l. infection rates (**b**) and *Borrelia afzelii* infection rates (**c**) depending on the climate zone.

The overall TBP prevalence as well as *B. burgdorferi* s.l. infections had the highest median incidence rates in the subtropical climate zone. *B. afzelii* infections had the highest median incidence rate in the cold temperate climate zone (Table 2, Fig. 6).

**Table 2:** Comparison of the overall tick-borne pathogen (TBP) prevalence and *Borrelia burgdorferi* s.l. and *Borrelia afzelii* infections in *Ixodes ricinus* ticks according to climate zone.

Category	Variable	p value
Temperate warm-subtropical	Overall TBP prevalence	0.002
	*B. burgdorferi* s.l. infections	0.001
Temperate warm-temperate cold	*B. afzelii* infections	0.041
Temperate cold-subtropical	*B. burgdorferi* s.l. infections	0.001

## Discussion

Our retrospective analysis revealed that infections in *I. ricinus* ticks from urban and suburban landscapes are influenced by geographic conditions. The most important aspects contributing to the increasing number of tick bites in cities are climate change and the exponential growth of agglomerations, which are taking over the natural habitats of ticks (historically, forests and meadows)^4,19^. It is worth emphasizing once again that tick bites may be related to an increased risk of contracting TBD, such as Lyme borreliosis, anaplasmosis, tick-borne encephalitis (TBE), babesiosis and rickettsiosis^4,15^.

We gathered 53 scientific reports of *I. ricinus* tick infections with various TBPs; the ticks in these reports were collected from urban and suburban areas in Europe over more than 20 years. It is worth emphasizing that over the years, molecular biology methods have developed substantially. Currently, we are equipped with technical possibilities allowing the detection of new pathogens, even those previously unknown. Our goal was to review the data, which covered a long timeframe (1991 to 2017). The literature presents changes in the diagnostic techniques used in molecular biology, which we should be aware of when analysing the results.

The results did not show any statistically significant differences in the prevalence of TBP between Northern and Southern Europe, presumably because of the small sample sizes from both regions.

The analysis showed a greater risk of *A. phagocytophilum* infections in Eastern Europe than in Western Europe. Similar infection rates in *I. ricinus* ticks were reported in studies from the Czech Republic (0.8–7.2%)^17,51^, Hungary (8.8%)^11^, Poland (1.7–14.0%)^1,43,44^, Slovakia (2.9–7.2%)^29,46,57^ and the Ukraine (5.2%)^58^. Significantly lower infection rates were observed in Western Europe, e.g., Switzerland (1.4–1.5%)^18,27^ and Germany (1.8–4.4%)^19,23–25,35,50^. However, two reports from Germany presented contradictory results, the origin of which is unexplainable with the present data and should therefore be further investigated^36,39^. The results obtained in this study may reflect host availability for *A. phagocytophilum*. This bacterium is known to infect a variety of different animals, including small mammals, wild boars, red foxes, ruminants and birds^4,19,36,64^. As *A. phagocytophilum* is not transmitted transovarially, reservoir hosts are crucial for the completion of its life cycle^19,64^. It is also important to note that many *A. phagocytophilum* strains occurring in Europe are nonpathogenic to humans^64,65^. Nevertheless, anaplasmosis is considered an emerging pathogen. Huhn *et al*. suggested that it is possible that human granulocytic anaplasmosis (HGA) is underdiagnosed in Europe^64^.

In this study, the median *Ca*. Neoehrlichia mikurensis infection rate was greater in Western Europe than in Eastern Europe. In an extensive study conducted by Oechslin *et al*. in Switzerland, the authors reported an overall *Ca*. Neoehrlichia mikurensis prevalence of 6.2%^27^. Similar results were obtained in the same country by Lommano *et al*. (6.4%)^18^. A much lower percentage of infected ticks was observed in research performed in urban areas of Eastern Europe: Slovakia (0.9–2.7%)^29,45,57^, Poland (0.2%)^1^ and the Czech Republic (2.2–3.0%)^51,57^. *Ca.* Neoehrlichia mikurensis is a newly emerging TBP. The first case of human infection was reported in 2010 in a Swedish patient who suffered from chronic leukocytic leukaemia and developed a prolonged fever, an erysipelas-like rash and thromboembolic complications^66^. A total of 18 cases of human infection with *Ca*. Neoehrlichia mikurensis have been reported in Europe to date^67^. This pathogen was discovered very recently; therefore, it is possible that it remains underdetected.

Ticks are sensitive to temperature and humidity changes. Along with other factors, such as host availability and density, climate conditions have a great impact on tick activity and infectivity throughout the year^60–62^. The results presented in the temperature and rainfall categories point to higher overall TBP prevalence in regions where the conditions are more conducive to tick growth. It is known that average temperatures over 0 °C in January and over 20 °C in July may favour longer activity throughout the year. However, during hot and dry summers, ticks stop questing and remain hidden at the ground level. Moreover, the comparison of climate zones showed the highest median incidence rate for overall TBP prevalence in the subtropical climate zone. Additionally, the results show a greater median incidence rate in Western Europe, presumably because of warmer weather conditions than in Eastern Europe^68^. Low temperatures prolong ticks’ developmental cycles and inhibit their host-seeking activities. Therefore, a warmer climate may contribute to lower mortality among ticks and enable the establishment of permanent populations as well as faster tick development^60,69^. Temperatures that are too high may lead to decreased humidity in the environment, causing desiccation from water loss^60,61,70^.

In this study, the median overall TBP prevalence was higher in areas with an average January rainfall over 50 mm. Although winter conditions may contribute to higher mortality and prolonged development among ticks, snow covering the ground for a long period of time protects overwintering ticks by providing protection from low temperatures^61^. Higher rainfall may also contribute to the maintenance of sufficient soil humidity during the drier times of the year, which, along with good cover of vegetation, is crucial for *I. ricinus* in their non-parasitic phases^71^. For average July rainfall, the results show that the median overall TBP prevalence was greater in regions with lower levels of precipitation. Although such conditions are generally favourable to tick activity^72^, prolonged dry weather combined with high temperatures may result in increased tick mortality^71,72^. However, studies conducted in Ireland show that *I. ricinus* ticks will quest under such conditions as long as they can rehydrate under proper vegetation cover^73^.

The division of data based on the climate zone revealed greater medians for *B. burgdorferi* s.l. infections in the subtropical and warm temperate climate zones. This result is supported by the fact that the pathogen reached a higher median in regions with average temperatures in July over 20 °C. Moreover, the distribution of Lyme borreliosis by *Ixodes* ticks is limited by a temperature range between −10 °C and +35 °C^60^. Other requirements for transmission are proper host availability and constant relative humidity higher than 80%^60,71^. Interestingly, the median incidence rate of *B. afzelii* infections was the highest in the cold temperate zone. *Borrelia garinii* and *B. burgdorferi* s.s. are quite frequent in Central and Western Europe, yet many studies suggest that *B. afzelii* is the most common pathogen among *B. burgdorferi* s.l. Grygorczuk *et al*. investigated the prevalence of the *B. burgdorferi* s.l. genospecies in a group of patients with different clinical forms and stages of Lyme borreliosis in northeastern Poland and concluded that *B. afzelii* was the dominant pathogen, followed by *B. garinii* and *B. burgdorferi* s.s.^74^. Additionally, Stanek *et al*. noticed the variations in the geographic distributions and clinical manifestations of Lyme disease for each species. In Europe, the infection is predominantly caused by *B. afzelii*, which usually remains localized to the skin, and *B. garinii*, which is usually associated with the nervous system^75^_._

In conclusion, our retrospective analysis revealed many differences in the infections of ticks collected from urban and suburban landscapes across various categories. A summary of the available results from the research on this subject revealed that the geographical distributions of ticks as well as climate conditions are related to the prevalence of different pathogens. It is important to conduct research on ticks in cities, as the prevalence of pathogens carried by ticks in urban areas is still not well known, despite ever-growing interest. Knowledge of the geographical distributions of ticks and pathogens may support assessments of the risk of infection and improve the diagnosis and treatment of patients.

## Supplementary information

Supplementary Table S1.

## Data Availability

The datasets generated and/or analysed during this study are available from the corresponding author upon reasonable request.
