# Supplementary material for: Comparison of tick-borne pathogen prevalence in Ixodes ricinus ticks collected in urban areas of Europe
Source: Sci Rep. 2020 Apr 24;10:6975. doi: 10.1038/s41598-020-63883-y (PMC7181685; doi:10.1038/s41598-020-63883-y)
Supplement: Supplementary file 1 — Supplementary Table S1. [file 41598_2020_63883_MOESM1_ESM.docx]

**Comparison of tick-borne pathogens prevalence in *Ixodes ricinus* ticks collected in urban areas of Europe**

Anna Grochowska^a^, Robert Milewski^b^, Sławomir Pancewicz^a^, Justyna Dunaj^a^, Piotr Czupryna^a^, Anna Justyna Milewska^b^, Magdalena Róg-Makal^c^, Sambor Grygorczuk^a^, and Anna Moniuszko-Malinowska^a^

^a^ Department of Infectious Diseases and Neuroinfections, Medical University of Białystok, Żurawia 14 15-540, Białystok, Poland

^b^ Department of Statistics and Medical Informatics, Medical University of Białystok, Szpitalna 37, Białystok, Poland

^c^ Department of Invasive Cardiology, Medical University in Białystok, M. Skłodowskiej-Curie 24A, 15-276, Białystok, Poland

**Corresponding Author:**

Anna Grochowska

Department of Infectious Diseases and Neuroinfections

Medical University of Bialystok, Żurawia 14

15-540 Bialystok, Poland

Tel.: 00 48 85 740 95 14; fax: 00 48 85 740 95 15;

E-mail: matosek.ania@gmail.com

Supplementary Table S1

Occurrence of tick-borne pathogens in questing *Ixodes ricinus* ticks from urban and suburban areas of Europe.

| Country | City | No. examined ticks | Overall tick-borne pathogens (TBP) prevalence (%) | Pathogens present (%/No. examined ticks) | | | | | | | | | | | | | | | | | | | | | | | | | | References |
| --- | --- | --- | --- | --- | --- | --- | --- | --- | --- | --- | --- | --- | --- | --- | --- | --- | --- | --- | --- | --- | --- | --- | --- | --- | --- | --- | --- | --- | --- | --- |
| Czech Republic | Prague (U), 2007 | 278 (60 F, 59 M, 159 N) | 4.7 F, 6.5 M, 17 N | Bsl (3.6 F, 1.8 M, 15.5 N) | | | | | | | | | | | | | Ap (1.1 F, 4.7 M, 1.4 N) | | | | | | | | | | | | | ^17^ |
|  | Stupno (S), 2007 | 123 (8 F, 10 M, 105 N) | 1.7 F, 8.1 N | Bsl (1.7 F, 7.3 N) | | | | | | | | | | | | | Ap (0.8 N) | | | | | | | | | | | | | ^17^ |
|  | Valtice (U), 2011-2014 | 968 (382 A, 586 N) | 7.9 A, 7.2 N | Bab (0.2 A, 0.2 N), | | | | | | Rick (2.3 A, 3.5 N) | | | | | | | Ap (3.7 A, 2.2 N) | | | | | | | Cm (1.7 A, 1.3 N) | | | | | | ^51^ |
|  | Praga, Park Stromovka (U), 1995-1998 | 779 (207 A, 572 N) | 3.2 A,  1.3 N | Bsl (3.2 A, 1.3 N) | | | | | | | | | | | | | | | | | | | | | | | | | | ^8^ |
|  | Brno (U), 1992 | 163 (64 F, 65 M, 34 N) | 26.2 F,  19.5 M, 9.1 N | Bsl (26.2 F, 19.5 M, 9.1 N) | | | | | | | | | | | | | | | | | | | | | | | | | | ^12^ |
|  | Brno, Pisarky Park (S), 2002 | 305 (19 F, 22 M, 243 N) | 12.1 | Ba 4.6 | | | | | | Bg 4.9 | | | | | | | Bv 0.7 | | | | | | | Bss 0.7 | | | | | | ^31^ |
|  | Prague (U, S) 1994-1997 | 9120 (3500 F, 2893 M, 2727 N) | 6.8 | Bsl 6.8 | | | | | | | | | | | | | | | | | | | | | | | | | | ^32^ |
|  | Brno, Pisarky Park (S), 2005 | 598 | 8.4 | Bsl 8.4 | | | | | | | | | | | | | | | | | | | | | | | | | | ^54^ |
|  | Dvur Kralove (S) | 138 | 8.0 | Cm 2.2 | | | | | | | | | | | | | Am 5.8 | | | | | | | | | | | | | ^57^ |
| England | Salisbury (U), 2013-2014 | 349 N | 18,6 | Bsl 18.1^a^ | | | | | | | | | | | | | Bmi 5.7 | | | | | | | | | | | | | ^10^ |
|  | London (U), 2013 | 279 | 2,2 | Bsl 2.2 | | | | | | | | | | | | | | | | | | | | | | | | | | ^26^ |
|  | London (U parks) | 65 | 7.7 | Bsl 7.7 | | | | | | | | | | | | | | | | | | | | | | | | | | ^59^ |
| Finland | Helsinki (U, S) | 726 (189 F, 234 M, 303 N) | 32.2 F+M+N^b^ | Ba 13.7 | | | | | | Bg 5 | | | | | | | Bsl 0.7 | | | | | | | Ba+Bg 0.3 | | | | | | ^13^ |
| France | Senart Forest (S), 2008 | 558 (69 F, 129 M, 360 N) | 16.5 F, 12.5 M, 35 N | Bsl (3.9 F, 4.2 M, 6.6 N) | | | An (11.3 F, 6.5 M, 14.2 N) | | | | | Rick (0.9 F, 1.3 M, 2.3 N) | | | | | Bab (0.2 F, 0.4 M, 5.6 N) | | | | | Ft (0.2 F, 0.2 M) | | | | | Bt (5.6 N) | | | ^34^ |
| Germany | Hanover (U), 2015 | 2100 (288 F, 285 M, 1527 N) | 9 F, 8 M, 37.6 N | Rick (8 F, 7 M, 36 N) | | | | | | | | | | | | | Ap (1 F, 1 M, 1.6 N) | | | | | | | | | | | | | ^19^ |
|  | Hamburg (U), 2011 | 1400 (60 F, 81 M, 1259 N) | 90.2 | Bsl 34.1^c^ (1.6 F, 1.5 M, 31 N) | | | | | | | | Ap (0.01 F, 0.14 M, 3.4 N) | | | | | | | | | | Rick (2.6 F, 3 M, 46.9 N) | | | | | | | | ^23,24^ |
|  | Saarland and Rhineland-Palatinate (S) 2011-2012 | 3914 (671 F, 451 M, 2792 N) | 22.2 | Bsl (20.4) | | | | | | | | | | | | | Ap (1.8) | | | | | | | | | | | | | ^25^ |
|  | Bonn (u), 2003 | 385 A+N | 7.5 A, 11.2 N | Bsl (7.5 A, 11.2 N) | | | | | | | | | | | | | | | | | | | | | | | | | | ^20^ |
|  | Munich and Regensburg (U), 2011-2012 | 2800 (1836 A, 964 N) | 4.2 (3 A, 1.2 N) | Bab (0.3 A, 0.3 N) | | | | | | | | | | | | | Rick (2.8 A, 0.9 N) | | | | | | | | | | | | | ^28^ |
|  | Hanover (U), 2005 | 1646 (388 F, 389 M, 869 N) | 1.5 F, 1.2 M, 1.4 N | Ba (0.1 F, 0.1 N) | | | | | Bg (0.1 F, 0.1 M, 0.1 N) | | | | | | Bv (0.2 F, 0.1 M) | | | | Bss (0.1 F, 0.1 M, 0.1 N) | | | | | | Ap (1 F, 1 M, 1.2 N) | | | | | ^35^ |
|  | Bavaria region (U, S), 2009-2010 | 5569 | 9.0 | Ap (3.5 F, 3.9 M, 1.6 N) | | | | | | | | | | | | | | | | | | | | | | | | | | ^36^ |
|  | Bavaria region (U, S), 2009-2010 | 6593 | 0.4 | Bab 0.4 | | | | | | | | | | | | | | | | | | | | | | | | | | ^37^ |
|  | Bavaria region (U, S), 2009 | 4459 | 6.0 | Rick 6.0 | | | | | | | | | | | | | | | | | | | | | | | | | | ^37^ |
|  | Leipzig, Munich (U), Saarland (S), 2009 | 782 (297 F, 313 M, 172 N) | 14.7 | Bab 4.7 (1.9 F, 2.3 M, 0.5 N) | | | | | | | | | | | | | Ap 10.1 (3.7 F, 5.6 M, 0.8 N) | | | | | | | | | | | | | ^39^ |
|  | Hanover (U), 2010 | 2100 (176 F, 196 M, 1697 N, 31 L) | 30.7 (3 F, 3.2 M, 24.2 N, 0.3 L) | Ap (0.3 F, 0.5 M, 3.6 N, 0.04 L) | | | | | | | | | | | | | Rick (2.7 F, 2.7 M, 24.2 N, 0.2 L) | | | | | | | | | | | | | ^50^ |
| Hungary | Budapest (cemetery) (U), 2013 | 240 | 49.6 | Bsl 40.8 | | | | | | | | | | | | | Ap 8.8 | | | | | | | | | | | | | ^11^ |
|  | Budapest, Margaret Island (U), 2011-2012 | 534 (166 F, 214 M, 150 N, 4 L) | 42.5 | Rh (14.6 F, 8.4 M, 3.7 N) | | | | | | | | | | | | | Rick (7.5 F, 6.4 M, 2.6 N) | | | | | | | | | | | | | ^49^ |
| Italy | Rome – Insugherata Natural Reserve (U), 2011-2012 | 33 (22 F, 11 M) | 85 | Rick 70 | | | | | Eh 6 | | | | | | Cb 9 | | | | Bsl 27 | | | | | | Bm 12 | | | | | ^21,22^ |
|  | Imola (U), 2006 | 115 (9 A, 106 N) | 3.5 A, 66.6 N | Bsl (0.9 A, 9.6 N) | | | | | Rh (0.9 A, 1.7 N) | | | | | | Rm (13 N) | | | | Bth (1.7 A, 36.5 N) | | | | | | Btc (5.2 N) | | | | | ^56^ |
| Lithuania | Vilnius (U), 2005 | 36 A | 27.78 | Ba 18.8 | | | | | Bg 5.6 | | | | | | Em 2.8 | | | | Ba+Bg 2.8 | | | | | | Ba+Bg+Em 2.8 | | | | | ^55^ |
| Netherlands | Duin and Kruidberg (S), Bijlmerweide (Amsterdam) (U), 2000-2004 | 1088 | 13.8 | Bsl 7.8 | | | | | | | | An 5.1 | | | | | | | | | | Bsl+An 0.9 | | | | | | | | ^53^ |
| Poland | Wrocław – the Osobowicki Forest (S), 2009 | 108 (64 F, 44 M) | 20.4 F+M | Ba 10.2 | | | | | Bg 1.9 | | | | | | Bv 1.9 | | | | Bss 2.8 | | | | | | Bmi 6.5 | | | | | ^15^ |
|  | Warsaw (U), 2012-2015 | 2267 (1452 A, 1156 N) | 9.3 A, 1.6 N | Bsl/Bmi (9.3 A, 1.6 N) | | | | | | | | | | | | | | | | | | | | | | | | | | ^16^ |
|  | Gdansk, Sopot, Gdynia (U), 2009-2010 | 757 (219 F, 231 M, 307 N) | 6.2 | Bab (2.4 F, 1.5 M, 0.7 N) | | | | | | | | | | | | | Ap (0.4 F, 1.2 M, 0.1 N) | | | | | | | | | | | | | ^43^ |
|  | Łódź, Tomaszów Mazowiecki, Pułtusk, Warsaw, Węgrów, Biała Podlaska (U parks and S forests), 2009-2011 | 1175 | 16.9 | Ba 11.7 | | Bss 2.1 | | | | Bg 4.9 | | | | Bv 1.9 | | | Bss+Bg 1.1 | | | Bss+Bv 0.5 | | | | Bg+Bv 0.2 | | | | Bss+Bg+Bv 0.3 | | ^47^ |
|  | Warsaw (U forests), 2011 | 405 | 11.6 | Rick 7.7 | | | | | | Ap 3.0 | | | | | | | Cm 0.2 | | | | | | | Eh 0.7 | | | | | | ^1^ |
|  | Warsaw (U parks), 2009-2010 | 244 (81 F, 121 M, 33 N, 9 L) | 4.1 F, 3.8 M, 1.3 N | Bsl (3.7 F, 2.5 M) | | | | | | | | | | | | | Rick (0.4 F, 1.3 F, 1.3 N) | | | | | | | | | | | | | ^41^ |
|  | Gdynia, Gdańsk (U), 2008 | 1153 (426 F, 454 M, 273 N) | 1.6 | Bav 0.9 | | | | | | | | Bac 0.2 | | | | | | | | | | Bab 0.5 | | | | | | | | ^52^ |
|  | Warsaw, the Bielański Forest | 120 (41 F, 37 M, 42 N) | 9.2 F, 4.2 M, 5 N | Bsl (9.2 F, 4.2 M, 5 N) | | | | | | | | | | | | | | | | | | | | | | | | | | ^40^ |
|  | Gdańsk, Sopot, Gdynia (U, S), 2001 | 701 | 28.7 | Bsl 12.4 | | | | | | | | Ap 14.0 | | | | | | | | | | Bm 2.3 | | | | | | | | ^44^ |
|  | Płock, Warsaw, Siedlce, Biała Podlaska, Międzyrzec Podlaski (U, S), 2008-2009 | 628 | 23.4 | Bsl 15.0 | | | | | | | | Bth 6.5 | | | | | | | | | | Bth+Bsl 1.9 | | | | | | | | ^48^ |
| Russia | Moscow (U), 2006 | 76 (40 F, 36 M) | 17.1 F, 6.6 M | Ba 6.6 F | | | | | | | | Bg (9.2 F, 6.6 M) | | | | | | | | | | Bv 1.3 F | | | | | | | | ^14^ |
| Serbia | Belgrade (U), 1996-2005 | 6628 | 22.1 | Bsl 22.1 | | | | | | | | | | | | | | | | | | | | | | | | | | ^30^ |
|  | Novi Sad (U), 2004 | 222 (53 F, 21 M, 148 L) | 6.8 F, 1.4 M, 21.2 L | Bsl (6.8 F, 1.4 M, 21.2 L) | | | | | | | | | | | | | | | | | | | | | | | | | | ^33^ |
| Slovakia | Bratislava (U), Senec (S), Malacky (U), Kosice (U), Bardejov (S) | 979 | 5.5 | Cm 2.7 | | | | | | | | | | | | | Ap 2.9 | | | | | | | | | | | | | ^57^ |
|  | Bratislava (U), 2011-2013 | 2799 (584 F, 614 M, 1533 N) | 0.04 F, 0.4 M, 0.8 N | Bab (0.04 F, 0.4 M, 0.8 N) | | | | | | | | | | | | | | | | | | | | | | | | | | ^9^ |
|  | Bratislava (U), 2011-2013 | 2117 (358 F, 423 M, 1336 N) | 2.0 F, 2.0 M, 3.2 N | Ap (2.0 F, 2.0 M, 3.2 N) | | | | | | | | | | | | | | | | | | | | | | | | | | ^46^ |
|  | Bratislava (U), 2011-2013 | 2034 (347 F, 373 M, 1314 N) | 0.3 F, 0.1 M, 0.5 N | Cm (0.3 F, 0.1 M, 0.5 N) | | | | | | | | | | | | | | | | | | | | | | | | | | ^45^ |
|  | Kosice (U, S), 2010 | 491 | 16.9 | Cm 1.6 | | | | | | | | Ap 3.1 | | | | | | | | | | Bsl 12.2 | | | | | | | | ^29^ |
|  | Bratislava, Malacky, Martin (U, S), 2006-2011 | 1052 (246 F, 299 M, 507 N) | 2.4 F, 3.2 M, 3.9 N | Rick (2.4 F, 3.2 M, 3.9 N) | | | | | | | | | | | | | | | | | | | | | | | | | | ^42^ |
| Switzerland | Parks, cemeteries, river sides, suburban forests (U, S), 2016 | 1079 (135 F, 138 M, 740 N, 66 L) | 38.2 | Ba 8.2 | Bss 1.3 | | | Bg 2.8 | | | Bv 0.9 | | Bsl 2.3 | | | Bmi 2.5 | | Rh 13.2 | | | Rm 0.3 | | Ap 1.4 | | | Cm 6.2 | | | Bav 0.8 | ^27^ |
|  | Suburban forests (S), 2009-2010 | 1476 |  | Ap 1.5 | | Cm 6.4^d^ | | | | Rh 9.2 | | | | Rm 0.1 | | | Rick 0.8 | | | Bab 1.9 | | | | Ba 9.6 | | | | Bsl 0.7 | | ^18^ |
|  |  |  |  | Bg 6.2 | | Bv 4.0 | | | | Bss 1.4 | | | | Bb 1.2 | | | Bmi  1.0 | | | Bl 0.1 | | | | Bs 0.01 | | | |  | |  |
| Turkey | Istanbul (U), 2008 | 75 | 38.7 | Ba 1.3 | | | | | | Bg 9.3 | | | | | | | Bl 22.7 | | | | | | | Bv 5.3 | | | | | | ^38^ |
| Ukraine | Kiev (U), 2013-2014 | 696 (237 F, 287 M, 172 N) | 11.1 | Ba 3.9 | | | | | | Bg 0.1 | | | | | | | Bm 1.9 | | | | | | | Ap 5.2 | | | | | | ^58^ |

Abbreviations: Bsl – *Borrelia burgdorferi* s.l., Ba – *Borrelia afzelii*, Bg – *Borrelia garinii*, Bss – *Borrelia burgdorferi* sensu stricto, Bv – *Borrelia valaisiana*, Bl – *Borrelia lusitaniae*, Bs – *Borrelia spielmanii*, Bb – *Borrelia bavariensis*, Btu – *Borrelia turdi*, Bis – *Borrelia bisetti*, Bmi – *Borrelia miyamotoi*, Bab – *Babesia* spp., Bm – *Babesia microti*, Bav – *Babesia venatorum*, Bac – *Babesia canis*, Rick – *Rickettsia* spp., Rh – *Rickettsia helvetica*, Rm – *Rickettsia monacensis*, Ra – *Rickettsia amblyommi*, Rr – *Rickettsia raoultii*, Rc – *Rickettsia conorii*, An – *Anaplasma* spp., Ap – *Anaplasma phagocytophilum*, Eh – *Ehrlichia* spp., Ec – *Ehrlichia canis*, Em – *Ehrlichia muris*, Cm – *Candidatus* Neoehrlichia mikurensis, Cb – *Coxiella burnetti*, Bt – *Bartonella* spp., Bth – *Bartonella henselae*, Btc – *Bartonella clarridgeiae*, Ft – *Francisella tularensis*

^a^ Genospecies of *Borrelia burgdorferi* s.l. were identified in 52 out of 63 *Borrelia* positive ticks: Bg (54%), Bv (44.2%), Ba (1.9%).

^b^ Out of 234 positive ticks, 142 were typeable.

^c^ Genospecies of *Borrelia burgdorferi* s.l. were identified in 208 out of 477 *Borrelia* positive ticks: Bg (40.4%), Ba (33.7%), Bv (21.6%) Bss (11.1%), Bs (10%), Bis (3.4%), Bl (0.5%).

^d^ For *C.* Neoehrlichia mikurensis the number of tested ticks was 818.
